# Retraction: Emodin interferes with AKT1-mediated DNA damage and decreases resistance of breast cancer cells to doxorubicin

**DOI:** 10.3389/fonc.2023.1337635

**Published:** 2023-12-07

**Authors:** Frontiers Editorial Office

The journal retracts the February 2021 article cited above.

Following publication, the authors contacted the Editorial Office to request the retraction of the cited article, stating concerns regarding the use of an incorrect dataset in the published article. It was confirmed by the authors when they tried to repeat their experiments unsuccessfully. The findings and conclusions reported in the article are no longer supported by the data. An investigation was conducted in in accordance with Frontiers’ policies that confirmed this; therefore, the article is retracted.

This retraction was approved by the Chief Editors of Frontiers in Oncology and the Chief Executive Editor of Frontiers. The authors agree to this retraction.

